# Association between alcohol-induced osteonecrosis of femoral head and risk variants of *MMPS* in Han population based on a case-control study

**DOI:** 10.18632/oncotarget.16380

**Published:** 2017-03-18

**Authors:** Jianzhong Wang, Xugang Shi, Hua Yang, Jieli Du, Yongri Ouyang, Hong Wang, Tianbo Jin, Chao Chen

**Affiliations:** ^1^ National Engineering Research Center for Miniaturized Detection System, Northwest University, Xi'an, Shaanxi, China; ^2^ Department of Orthopedics and Traumatology, The 2nd affiliated Hospital of Inner Mongolia University, Hohhot, Inner Mongolia, China; ^3^ Xi'an Tiangen Precision Medical Research Institute, Xi'an, Shaanxi, China

**Keywords:** MMPs, alcohol-induced ONFH, SNPs, Han population

## Abstract

The study aimed to evaluate the association between *MMP* gene superfamily and alcohol-induced osteonecrosis of femoral head (alcohol-induced ONFH) risk given its high prevalence, poor therapeutic effect, and serious clinical prognosis. 308 subjects (mean age, 49.47 years; males, 64.0%) who participated in our control group and 300 alcohol-induced ONFH patients (mean age, 43.29 years; males, 99.7%) formed the case group was enrolled to estimate by statistical analysis. We selected 23 single nucleotide polymorphisms (SNPs) from *MMPs*, and performed the chi-squared test, Fisher's exact test, *t*-test and genetic model analyses. From the result, rs243849 which located in *MMP2* were 1.355 (1.014-1.811), 1.34 (1.01-1.78) in allele model and log-addictive model, respectively. And the *p*-value of rs243849 in Cochran-Armitage trend test is 0.044. Unfortunately, the similar results of these SNPs were not observed when adjusted by gender and age. Our study is not enough to supply a positive result to benefit for alcohol-induced ONFH clinical prevention, but guide out a new direction for further experiment.

## BACKGROUND

As one orthopedic clinical common disease, osteonecrosis of the femoral head (ONFH) is a disease of femoral bone marrow cells, fat cells and bone marrow cell degeneration and necrosis caused by different reasons, such as hip trauma, corticosteroid use, alcohol abuse, haemoglobinopathies, bone marrow transplantations, chemotherapy and exposure to radiation [1]. Depending on the different risk factors, we divided it into traumatic and non-traumatic [2]. Some studies found that the main pathogenesis of osteonecrosis is lipid metabolism abnormality [3]. In China, about 7 million people are the patient of osteonecrosis and annually have found 100-200 thousand new cases of ONFH [4]. Overall, the major patients are young with a long life requirement and excellent functional expectancy.

Excessive alcohol intake over a long period of time may lead to abnormal lipid metabolism in population which is the main risk factor of alcohol-induced ONFH [5, 6]. Some studies showed that the effect of alcohol on ONFH which induces bone marrow stromal cells differentiation into adipocytes. Alcohol induced a significant increase in serum triglyceride and cholesterol levels. Triglyceride deposition in osteocytes leads to pyknosis and an increased percentage of empty osteocyte lacunae that demonstrated intracellular lipid deposition ultimately lead to the death of osteocytes [1]. So far, *MMPs* is a matrix metalloproteinase (MMP) gene family that encoded more than 25 members of proteolytic enzymes family, and they need Ca2+, Zn2+ and other metal ions as auxiliary when they involve in physiological functions such as cell proliferation, apoptosis and angiogenesis [7]. These enzymes are essential to the process of extracellular matrix degradation. Some studies found that MMP2 (Matrix metalloproteinase-2) and MMP9 expressions suppressed under some pathological conditions which included lipid disturbance [8], and found MMP activity persistent increased in chronic injuries which likely induces the disruption of ultrastructure and tissue function of ECM (The Extracellular Matrix) [9]. As zinc-dependent endopeptidases, MMPs mainly digest collagen and other structural molecules. They directly involved in bone matrix degradation of bone metabolism. Bruni et al. [10] demonstrated that the result in a prostate cancer bone metastases research found varying degrees of osteolytic lesions in bone metastases site where active osteoclast proliferation significantly, and found MMP-9 as a proteolytic enzyme play a role in extracellular matrix degradation of osteoclasts. Viereck et al. [11] found that MMP-2 through the degradation of the bone matrix to start bone resorption and formation in the bone remodeling process. Previous studies indicated that MMPs were associated with bone metabolism disease and some chronic disease. However, the association between MMPs and alcohol-induced ONFH has been less researched.

With the in-depth molecular biology and genetics research of alcohol-induced ONFH, gene polymorphisms opened a new idea for us to study the association between them. In the study, we selected some polymorphisms in *MMPs* to explore the association with alcohol-induced ONFH in Han Chinese population.

## RESULTS

### Baseline characteristics

A total of 608 eligible subjects (300 patients, 308 healthy controls) were included in the study with the average age of 43.29±13.08 and 49.47±7.97 years. Baseline characteristics of the subjects were presented in Supplementary Table 1. From the results of independent samples test, we found age and gender exist statistics significant between case and control group.

### Association between MMPs SNPs and alcohol-induced ONFH risk

The basic information of 23 SNPs on *MMP2-MMP8* which we researched is enumerated in Table 1, including chromosome, position, band, allele, gene and MAF in case and control group. The primer of SNPs was designed by the Sequenom MassARRAY Assay Design 4.0 software [12]. Hardy-Weinberg equilibrium (HWE) which tested by PLINK used the genotype frequencies of control group proved all SNPs correspond with HWE. And the chi-square test results were shown in Table 2. From the result, we found rs243849 increased alcohol-induced ONFH risk (OR=1.36; 95%CI= 1.01-1.81; *p*=0.04). We from the dominant and recessive model Chi-square result hadn't found significant SNP.

**Table 1:** Basic information of SNPs examined in the *MMPs* genes

SNP rs#	Chr	Position	Band	Gene	Allele		Case				Control				Case		Control	Cochran-A Trend
					A	B	AA	AB	BB		AA	AB	BB		MAF		MAF	
rs14983	11	102391425	11q22.2	MMP7	A	G	20	122	158		20	121	167		0.270		0.261	0.730
rs17352054	11	102393055	11q22.2	MMP7	C	A	5	68	227		5	68	235		0.130		0.127	0.860
rs10502001	11	102398593	11q22.2	MMP7	T	C	20	122	158		20	121	166		0.270		0.262	0.756
rs11568818	11	102401661	11q22.2	MMP7	C	T	4	54	242		2	44	262		0.103		0.078	0.126
rs17098318	11	102402858	11q22.2	MMP7	A	G	4	53	243		2	43	263		0.102		0.076	0.125
rs3740938	11	102587062	11q22.2	MMP8	A	G	14	108	178		14	103	190		0.227		0.213	0.573
rs2012390	11	102590777	11q22.2	MMP8	G	A	20	114	165		19	113	176		0.258		0.245	0.620
rs1940475	11	102593248	11q22.2	MMP8	T	C	43	132	125		35	144	129		0.363		0.347	0.564
rs11225394	11	102595413	11q22.2	MMP8	T	C	0	65	235		0	53	238		0.108		0.091	0.294
rs11225395	11	102596480	11q22.2	MMP8	A	G	40	131	129		34	143	131		0.352		0.343	0.739
rs639752	11	102707339	11q22.2	MMP3	C	A	26	135	139		32	146	130		0.312		0.341	0.264
rs650108	11	102708787	11q22.2	MMP3	G	A	47	140	112		47	166	94		0.391		0.423	0.244
rs520540	11	102709425	11q22.2	MMP3	A	G	26	135	139		32	146	130		0.312		0.341	0.264
rs646910	11	102709522	11q22.2	MMP3	A	T	2	42	256		2	46	260		0.077		0.081	0.772
rs602128	11	102713465	11q22.2	MMP3	A	G	26	136	137		32	143	130		0.314		0.339	0.343
rs679620	11	102713620	11q22.2	MMP3	T	C	26	136	138		33	145	130		0.313		0.343	0.266
rs678815	11	102713777	11q22.2	MMP3	G	C	26	134	137		32	146	130		0.313		0.341	0.291
rs522616	11	102715048	11q22.2	MMP3	C	T	49	138	113		40	144	123		0.393		0.365	0.309
rs1053605	16	55519607	16q12.2	MMP2	T	C	6	62	232		3	63	242		0.123		0.112	0.542
rs243849	16	55523705	16q12.2	MMP2	T	C	17	92	191		9	83	216		0.210		0.164	0.045
rs243847	16	55523998	16q12.2	MMP2	C	T	48	134	118		59	140	109		0.383		0.419	0.221
rs243832	16	55539191	16q12.2	MMP2	C	G	34	150	116		41	145	122		0.363		0.369	0.848
rs7201	16	55539614	16q12.2	MMP2	C	A	12	110	178		18	116	173		0.223		0.248	0.312

**Table 2:** The HWE and Chi-square test results in allele and genotype model of SNPs

SNP ID	Allele		HWE	Allele			Genotype	Dominant	Recessive
	A	B	p	OR	95% CI	p	p	p	p
rs14983	A	G	0.8827	1.05	0.81-1.35	0.73	0.93	0.70	0.93
rs17352054	C	A	1	1.03	0.74-1.44	0.86	0.98	0.86	0.78
rs10502001	T	C	0.8826	1.04	0.81-1.34	0.76	0.94	0.73	0.94
rs11568818	C	T	0.7036	1.36	0.92-2.02	0.12	0.35	0.15	0.66
rs17098318	A	G	0.6915	1.37	0.92-2.04	0.12	0.35	0.15	0.66
rs3740938	A	G	1	1.08	0.82-1.42	0.58	0.81	0.52	0.95
rs2012390	G	A	0.8779	1.07	0.82-1.38	0.62	0.88	0.63	0.79
rs1940475	T	C	0.6175	1.07	0.85-1.36	0.56	0.52	0.96	0.27
rs11225394	T	C	0.1469	1.21	0.83-1.78	0.32	0.31	0.29	1.00
rs11225395	A	G	0.7041	1.04	0.82-1.32	0.74	0.63	0.91	0.39
rs639752	C	A	0.3762	0.88	0.69-1.11	0.28	0.54	0.31	0.47
rs650108	G	A	0.07913	0.88	0.7-1.1	0.25	0.16	0.08	0.89
rs520540	A	G	0.3762	0.88	0.69-1.11	0.28	0.54	0.31	0.47
rs646910	A	T	1	0.94	0.62-1.43	0.77	0.93	0.75	0.63
rs602128	A	G	0.5228	0.89	0.7-1.14	0.36	0.63	0.43	0.45
rs679620	T	C	0.5267	0.88	0.69-1.11	0.28	0.53	0.35	0.39
rs678815	G	C	0.3762	0.88	0.69-1.12	0.30	0.57	0.33	0.49
rs522616	C	T	0.9022	1.13	0.9-1.42	0.31	0.50	0.54	0.25
rs1053605	T	C	0.7803	1.12	0.79-1.58	0.54	0.58	0.71	0.48
rs243849	T	C	0.6826	1.36	1.01-1.81	0.04	0.11	0.09	0.09
rs243847	C	T	0.2434	0.86	0.69-1.09	0.21	0.47	0.31	0.31
rs243832	C	G	0.9028	0.98	0.77-1.24	0.85	0.68	0.81	0.46
rs7201	C	A	0.879	0.87	0.67-1.14	0.32	0.51	0.46	0.29

Values in bold indicate the SNP, which was significant correlated with alcohol-induced ONFH.

Subsequently, we performed unconditional logistic regression model to calculate the risk allele which using the allele frequencies of the lowest-risk allele in control group. The comparative advantage, odds ratios (OR) and 95% confidence intervals (CI) to assess the association between SNPs in *MMPs* and alcohol-induced ONFH risk in five genetic models which including codominant, dominant, recessive, overdominant and additive model. The results of SNPs in MMP2 shown in Table 3 and others which were not significant shown in Supplementary Table 2. Rs243849 was significant increased alcohol-induced ONFH risk in log-additive model (OR:1.34; 95%: 1.01-1.78; *p*=0.044). After adjustment gender and age, we found no SNP exist statistically significant (Supplementary Table 3).

**Table 3:** Logistic analyses of SNPs association with alcohol-induced ONFH in without adjustment addition

SNP	Model	Genotype	Control	Alcohol	OR (95% CI)	p
rs1053605	Codominant	C/C	242 (78.6%)	232 (77.3%)	1	0.57
		C/T	63 (20.4%)	62 (20.7%)	1.03 (0.69-1.52)	
		T/T	3 (1%)	6 (2%)	2.09 (0.52-8.44)	
	Dominant	C/C	242 (78.6%)	232 (77.3%)	1	0.71
		C/T-T/T	66 (21.4%)	68 (22.7%)	1.07 (0.73-1.58)	
	Recessive	C/C-C/T	305 (99%)	294 (98%)	1	0.29
		T/T	3 (1%)	6 (2%)	2.07 (0.51-8.37)	
	Overdominant	C/C-T/T	245 (79.5%)	238 (79.3%)	1	0.95
		C/T	63 (20.4%)	62 (20.7%)	1.01 (0.68-1.50)	
	Log-additive	---	---	---	1.11 (0.79-1.58)	0.54
rs243849	Codominant	C/C	216 (70.1%)	191 (63.7%)	1	0.11
		C/T	83 (26.9%)	92 (30.7%)	1.25 (0.88-1.79)	
		T/T	9 (2.9%)	17 (5.7%)	2.14 (0.93-4.90)	
	Dominant	C/C	216 (70.1%)	191 (63.7%)	1	0.09
		C/T-T/T	92 (29.9%)	109 (36.3%)	1.34 (0.95-1.88)	
	Recessive	C/C-C/T	299 (97.1%)	283 (94.3%)	1	0.092
		T/T	9 (2.9%)	17 (5.7%)	2.00 (0.88-4.55)	
	Overdominant	C/C-T/T	225 (73%)	208 (69.3%)	1	0.31
		C/T	83 (26.9%)	92 (30.7%)	1.20 (0.84-1.70)	
	Log-additive	---	---	---	1.34 (1.01-1.78)	0.044
rs243847	Codominant	T/T	109 (35.4%)	118 (39.3%)	1	0.47
		C/T	140 (45.5%)	134 (44.7%)	0.88 (0.62-1.26)	
		C/C	59 (19.2%)	48 (16%)	0.75 (0.47-1.19)	
	Dominant	T/T	109 (35.4%)	118 (39.3%)	1	0.31
		C/T-C/C	199 (64.6%)	182 (60.7%)	0.84 (0.61-1.17)	
	Recessive	T/T-C/T	249 (80.8%)	252 (84%)	1	0.31
		C/C	59 (19.2%)	48 (16%)	0.80 (0.53-1.22)	
	Overdominant	T/T-C/C	168 (54.5%)	166 (55.3%)	1	0.85
		C/T	140 (45.5%)	134 (44.7%)	0.97 (0.70-1.33)	
	Log-additive	---	---	---	0.87 (0.70-1.09)	0.22
rs243832	Codominant	G/G	122 (39.6%)	116 (38.7%)	1	0.68
		G/C	145 (47.1%)	150 (50%)	1.09 (0.77-1.53)	
		C/C	41 (13.3%)	34 (11.3%)	0.87 (0.52-1.47)	
	Dominant	G/G	122 (39.6%)	116 (38.7%)	1	0.81
		G/C-C/C	186 (60.4%)	184 (61.3%)	1.04 (0.75-1.44)	
	Recessive	G/G-G/C	267 (86.7%)	266 (88.7%)	1	0.46
		C/C	41 (13.3%)	34 (11.3%)	0.83 (0.51-1.35)	
	Overdominant	G/G-C/C	163 (52.9%)	150 (50%)	1	0.47
		G/C	145 (47.1%)	150 (50%)	1.12 (0.82-1.55)	
	Log-additive	---	---	---	0.98 (0.77-1.24)	0.85
rs7201	Codominant	A/A	173 (56.4%)	178 (59.3%)	1	0.51
		C/A	116 (37.8%)	110 (36.7%)	0.92 (0.66-1.29)	
		C/C	18 (5.9%)	12 (4%)	0.65 (0.30-1.39)	
	Dominant	A/A	173 (56.4%)	178 (59.3%)	1	0.46
		C/A-C/C	134 (43.6%)	122 (40.7%)	0.88 (0.64-1.22)	
	Recessive	A/A-C/A	289 (94.1%)	288 (96%)	1	0.29
		C/C	18 (5.9%)	12 (4%)	0.67 (0.32-1.41)	
	Overdominant	A/A-C/C	191 (62.2%)	190 (63.3%)	1	0.78
		C/A	116 (37.8%)	110 (36.7%)	0.95 (0.69-1.32)	
	Log-additive	---	---	---	0.87 (0.66-1.14)	0.31

Values in bold indicate that the locus has statistically significant.

### Haplotype analysis

**Figure 1:**
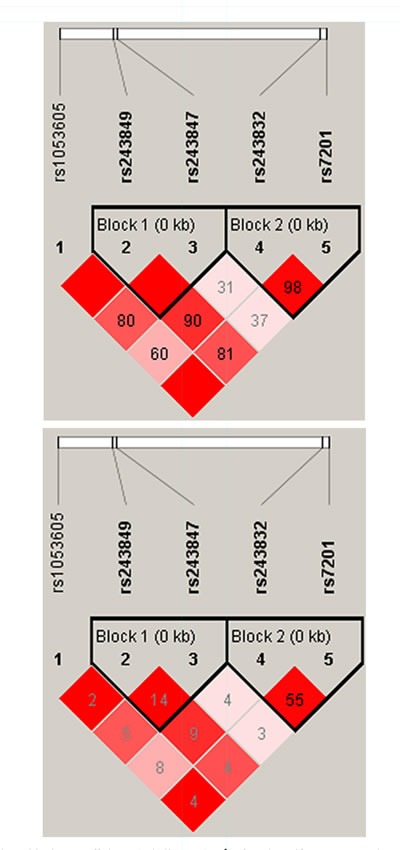
Linkage disequilibrium coefficients (a, |*D*’|) and (b, r2) of the identified haplotype in the MMP2 gene

We using parameter D’ and r2 to measure the extent of linkage disequilibrium between SNPs, and according to the control group data to determine the haplotype LD block. From the Pearson Chi-square results, we found the haplotype block consisted by rs243849 and rs243847 exist statistically significant (*p*=0.039) (Figure 1 and Table 4). The result of the unconditional logistic regression model, we didn't find a significant association between different haplotype blocks and alcohol-induced ONFH.

**Table 4:** Haplotype association with response of rs243849 and rs243847

Haplotype	Freq.	Case, Control Ratio Counts	Case, Control Fre	Chi	p
CT	0.412	244.0 : 356.0, 257.0 : 359.0	0.407, 0.417	0.139	0.709
CC	0.401	230.0 : 370.0, 258.0 : 358.0	0.383, 0.419	1.594	0.207
TT	0.187	126.0 : 474.0, 101.0 : 515.0	0.210, 0.164	4.243	0.039

## DISCUSSION

ONFH is a kind of multi-factor and multi-pathology process and is a disease with the outcome of the femoral head weight-bearing area of osteonecrosis. A variety of environmental factors and the complex regulatory network of body affect the occurrence and development of ONFH, such as trauma, alcohol abuse, hormone abuse, smoking, lipid metabolism disorders, fibrinolytic system disorders, cell apoptosis, antioxidant enzymes, bone marrow stem cell damage and genetic diseases. The special and complex distribution of blood vessels in the femoral head and hip joints is the most important part of the load in body, so that various risk factors easily lead to femoral head ischemia. Although there are many causes of ONFH, its pathology process is basically the same. The theory of blood supply damage in the weight-bearing area of the femoral head always dominant in the research of ONFH. There are other theories of the pathogenesis of ONFH, such as the theory of intravascular coagulation abnormalities, osteoblast and osteocyte apoptosis and bone cell fat necrosis.

Recently, the amount of study researches ONFH related gene SNP hereditary molecular etiology to clarify the genetic molecular mechanism of ONFH. Bjorkman A et.al for the first time proved that the blood coagulation factor V (G1691A) is positively correlated with ONFH [13, 14]. The research of Kim et.al verified that rs2227631, rs1799889, and rs11178 which located in *PAL-1*(Plasminogen-activating inhibitor-1) are significantly associated with the risk of ONFH [15]. Zhang et.al researched that association between *eNOS* with ONFH, found G894T in *eNOS* is associated with ONFH risk [16]. Amount of researches explored the relationship between genetic polymorphism and ONFH risk, found a lot of gene including *ANXs*(annexins), *VEGF* (vascular endothelial growth factor), *HIF-1* (Hypoxia-inducible factor-1), Apolipoproteins B (*ApoB*) and apolipoproteins A1 (*ApoA1*) are associated with ONFH.

Scientists have found more than 20 different kinds of MMPs, proved that they can degrade the extracellular matrix. There are some studies proved that MMPs participates the process of bone metabolism and occupies a certain position in the pathology of some diseases, such as lipid metabolic disorders, inflammation and malignant [17–19]. In our study, we only found rs243849 which located in MMP2 were significant with increased alcohol-induced ONFH in without adjustment models and the association is not significant after adjustment. We found age and gender are the effective confounding factors in case and control group. After adjustment, the association between SNPs and alcohol-induced ONFH were not significant, which might be partly attributed to sample size that is still small and gender distribution that is unbalanced for female.

In normal physiological conditions, as a complicated process that human bone metabolism including two important parts: bone resorption and bone formation. The interaction among bone cells, blood cells, and stroma cells consists of the metabolism process of bone. Two kinds of cells including osteoclasts and osteoblasts which differentiated from bone marrow stroma cells (BMSCs) play an important role in the metabolism process of bone [20]. The process of osteoblasts acts in the compound bone matrix and osteoclasts participates in bone resorption preserve a dynamic balance to maintain the normal physiological state of bone. If the dynamic equilibrium of bone metabolism lost, orthopedic diseases will occur.

Some previous researches confirmed that the good osteogenic function of BMSCs is existence about patients with alcohol-induced ONFH [21, 22]. Our research aimed to explore the affection power of genetic factors for alcohol-induced ONFH. MMPs is secreted by osteoblasts and activate osteoclasts meantime. We explored the function of 23 SNPs located in *MMP2—MMP8* to detect the mechanism of alcohol-induced ONFH. The expression of MMP2 and MMP7 were significantly upregulated when patients with ONFH, which means the decreased capacity of repair capacity and altered bone remodeling [23]. In the research of Seiya Jingushi also found MMP3 is increased in the disease process, further know that the concentration of MMP3 is higher in early stage disease than in later stage disease [24]. MMPs overexpressed may accelerate the apoptosis of bone and bone matrix degradation and promote bone resorption which leads to bone trabecular thinning, osteoporosis, atrophy or broken bone cannot withstand normal stress load [25, 26]. Bone trabecular microfracture led to microvascular damage and trabecular micro-circulation interruption which combined with lipid metabolism disorders and abnormal blood clotting mechanisms lead to ONFH. Based on the differences of osteogenic and adipogenic differentiation, one study found that the BMSCs adipocytes capability of patients with alcohol-induced ONFH higher than femoral neck fracture patients, however, found osteogenic differentiation function is below the femoral neck fracture [27, 28]. That further evidence the altered differentiation function may be one of the pathogenesis of alcohol-induced ONFH.

Rico et al. reported that alcohol-induced ONFH induced by alcohol stimulate the excessive secretion of adrenal glucocorticoids, and found that alcohol and hormones may stimulate ONFH through a common pathway [29, 30]. Alcohol caused lipid metabolism the abnormal increase of fatty substance in blood circulation which gathered into a ball of fat, lead to reduced blood flow speed, clogging capillaries and causing the femoral head microvascular thrombosis, eventually leading to ONFH [31, 32].

From the above, we first obtained one hypothesis that alcohol like the mechanism of a hormone that directly induces MMPs gene express to change the BMSCs differentiation direction which caused BMSCs differentiate into adipocytes and osteoclasts, reduced its osteoblast differentiation. And on the other hand, the abnormal lipid metabolism is the other pathology mechanism of alcohol-induced ONFH.

## CONCLUSIONS

We only found rs243849 was significant increased alcohol-induced ONFH risk in allele and log-additive model. But after adjustment, the result was not further verified. The limitations of our study should be mentioned. All the samples that were from Han population who lived in Zhengzhou city and around area. The confounding factors of the population may cause type I error (false positive). The gender ratio imbalance may be the other factor which leads to the not statistically significant results.

## MATERIALS AND METHODS

### Ethics statement

The research protocol was performed according the principles of the Declaration of Helsinki and was consented by the Medical Ethical Committee of Zhengzhou Traditional Chinese Medicine Traumatology Hospital. In the initial stage, the recruited participants agreed to provide baseline blood samples and questionnaire information. Written informed consent was obtained from all volunteers and study protocols were performed in accordance with the regular guideline.

### Study participants

The study subjects were derived from Zhengzhou Traditional Chinese Medicine Traumatology Hospital up to January 2016 from September 2014 which recruited a total of 608 individuals including 300 patients and 308 healthy controls for our case-control study. These controls did not have osteonecrosis and other related diseases. They were all Han Chinese and lived in Zhengzhou city or the surrounding area. We selected them from September 2014 to January 2016 in Zhengzhou Traditional Chinese Medicine Traumatology Hospital. Combined the normal epidemiological investigation, the sample of case group were chosen based on the clinical manifestations such as hip pain, activity limitation of hip, lower limb muscle atrophy of the sick side, and combined with the videography examination, higher density shadows, rupture of the joint surface, or bumpiness and narrowness of the hip joint etc.. When patients without X-ray changed, magnetic resonance imaging (MRI) was used to make a definite diagnosis. The exclusion criteria were: (1) Individuals suffering from serious primary diseases and required steroid treatment for replacement. (2) It did not satisfy the diagnostic criteria of alcohol-induced ONFH, patients diagnosed with traumatic osteonecrosis or other hip diseases. (3) Patients affected by drugs that cause liver disease or dyslipidemia.

### SNP selection and genotyping

We selected these 23 validated SNPs which had an MAF > 5% in the HapMap Asian population (CHB and JPT). Based on the public databases: dbSNP (https://www.ncbi.nlm.nih.gov/projects/SNP/) and HapMap (http://www.hapmap.org/), SNPs was selected from these dense tagging of SNPs which has the linkage disequilibrium (LD) blocks within *MMPs* gene and deleted some loci which call rate <0.9 in the experiment. Venous blood samples (5 mL) were collected from every person in our examination. DNA was extracted from whole blood samples using a genomic DNA purification kit (GoldMag, China), and the blood was stored with a condition of −20°C. The DNA concentration was measured by spectrometry (DU530 UV/VIS spectrophotometer, Beckman Instruments, Fullerton, CA, USA). The Sequenom MassARRAY Assay Design 4.0 software (Sequenom, Inc, San Diego, CA, USA) was used to design the Multiplexed SNP Mass EXTEND assay [12]. Genotyping *MMPS* SNPs were performed using a Sequenom MassARRAY RS1000 (Sequenom, Inc.) according to the standard protocol.

### Statistical analysis

The SequenomTyper 4.0 Software™ (Sequenom, Inc.) was used to manage and analyze the data. Categorical variables were presented as percentages (%) and continuous variables were expressed as mean± SD. We performed the comparison of variables using variance analysis for continuous variables and chi-square tests for categorical variables. The univariate and multivariable logistic regression models were using to examine the association between case and control, and corresponding odds ratios (ORs) and 95% confidence intervals (CIs) were calculated simultaneously. Potential confounders influencing the risk of alcohol-induced ONFH that were adjusted in the multivariable models were social-demographic variables (age, sex) that we knew and without other diseases history (coronary heart disease, diabetes, hypertension disease, tumor etc.). All statistical analysis was carried out using SPSS19.0 statistical software (SPSS, Chicago, IL) and Microsoft Excel, and a two-tailed *P* value of <0.05 was considered statistically significant. PLINK software (http://pngu.mgh.harvard.edu/purcell/plink/) was used by four models (dominant, recessive, codominant and log-additive models) to evaluate the case-control association about 23 SNPs and alcohol-induced ONFH risk. Lastly, Haploview software (version 4.2) was performed to analyses Linkage disequilibrium (LD).

## SUPPLEMENTARY MATERIALS TABLES







## Abbreviations

MMP: matrix metalloproteinase
alcohol-induced ONFH: alcohol-induced osteonecrosis of femoral head
BMSC: bone marrow stroma cell
CIs: confidence intervals
HWE: Hardy-Weinberg equilibrium
MAF: minor allele frequency
ORs: odds ratios
SNPs: single nucleotide polymorphisms
ANXs: annexins
VEGF: vascular endothelial growth factor
HIF-1: Hypoxia-inducible factor-1
Apo: Apolipoproteins
