# Supplementary material for: Association between alcohol-induced osteonecrosis of femoral head and risk variants of MMPS in Han population based on a case-control study
Source: Oncotarget. 2017 Mar 18;8(38):64490–8. doi: 10.18632/oncotarget.16380 (PMC5610020; doi:10.18632/oncotarget.16380)
Supplement: Supplementary file 1 [file oncotarget-08-64490-s001.docx]

Association between alcohol-induced osteonecrosis of femoral head and risk variants of *MMPS* in Han population based on a case-control study

**Supplementary Material**

Supplementary Table 1. Basic character of the study population.

|  |  | Gender | | Total | Age | |
| --- | --- | --- | --- | --- | --- | --- |
|  |  | female | male |  | Mean | Std. Deviation |
| Group | Alcohol | 1 | 299 | 300 | 43.29 | 13.084 |
|  | control | 111 | 197 | 308 | 49.47 | 7.973 |
| Total |  | 112 | 496 | 608 |  |  |
