# Supplementary material for: Association between alcohol-induced osteonecrosis of femoral head and risk variants of MMPS in Han population based on a case-control study
Source: Oncotarget. 2017 Mar 18;8(38):64490–8. doi: 10.18632/oncotarget.16380 (PMC5610020; doi:10.18632/oncotarget.16380)
Supplement: Supplementary file 2 [file oncotarget-08-64490-s002.docx]

Association between alcohol-induced osteonecrosis of femoral head and risk variants of *MMPS* in Han population based on a case-control study

**Supplementary Material**

Supplementary Table 2. Logistic analyses of SNPs in all research gene except MMP2 in crude analysis

| SNP | Model | Genotype | Control | Alcohol | OR (95% CI) | *p* |
| --- | --- | --- | --- | --- | --- | --- |
| rs14983 | Codominant | G/G | 167 (54.2%) | 158 (52.7%) | 1 | 0.93 |
|  |  | G/A | 121 (39.3%) | 122 (40.7%) | 1.07 (0.76-1.49) |  |
|  |  | A/A | 20 (6.5%) | 20 (6.7%) | 1.06 (0.55-2.04) |  |
|  | Dominant | G/G | 167 (54.2%) | 158 (52.7%) | 1 | 0.7 |
|  |  | G/A-A/A | 141 (45.8%) | 142 (47.3%) | 1.06 (0.77-1.46) |  |
|  | Recessive | G/G-G/A | 288 (93.5%) | 280 (93.3%) | 1 | 0.93 |
|  |  | A/A | 20 (6.5%) | 20 (6.7%) | 1.03 (0.54-1.95) |  |
|  | Overdominant | G/G-A/A | 187 (60.7%) | 178 (59.3%) | 1 | 0.73 |
|  |  | G/A | 121 (39.3%) | 122 (40.7%) | 1.06 (0.77-1.47) |  |
|  | Log-additive | --- | --- | --- | 1.05 (0.81-1.35) | 0.73 |
| rs17352054 | Codominant | A/A | 235 (76.3%) | 227 (75.7%) | 1 | 0.98 |
|  |  | C/A | 68 (22.1%) | 68 (22.7%) | 1.04 (0.71-1.52) |  |
|  |  | C/C | 5 (1.6%) | 5 (1.7%) | 1.04 (0.30-3.62) |  |
|  | Dominant | A/A | 235 (76.3%) | 227 (75.7%) | 1 | 0.86 |
|  |  | C/A-C/C | 73 (23.7%) | 73 (24.3%) | 1.04 (0.71-1.50) |  |
|  | Recessive | A/A-C/A | 303 (98.4%) | 295 (98.3%) | 1 | 0.97 |
|  |  | C/C | 5 (1.6%) | 5 (1.7%) | 1.03 (0.29-3.58) |  |
|  | Overdominant | A/A-C/C | 240 (77.9%) | 232 (77.3%) | 1 | 0.86 |
|  |  | C/A | 68 (22.1%) | 68 (22.7%) | 1.03 (0.71-1.52) |  |
|  | Log-additive | --- | --- | --- | 1.03 (0.74-1.44) | 0.86 |
| rs10502001 | Codominant | C/C | 166 (54.1%) | 158 (52.7%) | 1 | 0.94 |
|  |  | T/C | 121 (39.4%) | 122 (40.7%) | 1.06 (0.76-1.48) |  |
|  |  | T/T | 20 (6.5%) | 20 (6.7%) | 1.05 (0.54-2.03) |  |
|  | Dominant | C/C | 166 (54.1%) | 158 (52.7%) | 1 | 0.73 |
|  |  | T/C-T/T | 141 (45.9%) | 142 (47.3%) | 1.06 (0.77-1.46) |  |
|  | Recessive | C/C-T/C | 287 (93.5%) | 280 (93.3%) | 1 | 0.94 |
|  |  | T/T | 20 (6.5%) | 20 (6.7%) | 1.03 (0.54-1.95) |  |
|  | Overdominant | C/C-T/T | 186 (60.6%) | 178 (59.3%) | 1 | 0.75 |
|  |  | T/C | 121 (39.4%) | 122 (40.7%) | 1.05 (0.76-1.46) |  |
|  | Log-additive | --- | --- | --- | 1.04 (0.80-1.35) | 0.76 |
| rs11568818 | Codominant | T/T | 262 (85.1%) | 242 (80.7%) | 1 | 0.3 |
|  |  | C/T | 44 (14.3%) | 54 (18%) | 1.33 (0.86-2.05) |  |
|  |  | C/C | 2 (0.6%) | 4 (1.3%) | 2.17 (0.39-11.93) |  |
|  | Dominant | T/T | 262 (85.1%) | 242 (80.7%) | 1 | 0.15 |
|  |  | C/T-C/C | 46 (14.9%) | 58 (19.3%) | 1.37 (0.89-2.09) |  |
|  | Recessive | T/T-C/T | 306 (99.3%) | 296 (98.7%) | 1 | 0.39 |
|  |  | C/C | 2 (0.6%) | 4 (1.3%) | 2.07 (0.38-11.37) |  |
|  | Overdominant | T/T-C/C | 264 (85.7%) | 246 (82%) | 1 | 0.21 |
|  |  | C/T | 44 (14.3%) | 54 (18%) | 1.32 (0.85-2.03) |  |
|  | Log-additive | --- | --- | --- | 1.36 (0.92-2.01) | 0.13 |
| rs17098318 | Codominant | G/G | 263 (85.4%) | 243 (81%) | 1 | 0.3 |
|  |  | A/G | 43 (14%) | 53 (17.7%) | 1.33 (0.86-2.07) |  |
|  |  | A/A | 2 (0.6%) | 4 (1.3%) | 2.16 (0.39-11.92) |  |
|  | Dominant | G/G | 263 (85.4%) | 243 (81%) | 1 | 0.15 |
|  |  | A/G-A/A | 45 (14.6%) | 57 (19%) | 1.37 (0.89-2.10) |  |
|  | Recessive | G/G-A/G | 306 (99.3%) | 296 (98.7%) | 1 | 0.39 |
|  |  | A/A | 2 (0.6%) | 4 (1.3%) | 2.07 (0.38-11.37) |  |
|  | Overdominant | G/G-A/A | 265 (86%) | 247 (82.3%) | 1 | 0.21 |
|  |  | A/G | 43 (14%) | 53 (17.7%) | 1.32 (0.85-2.05) |  |
|  | Log-additive | --- | --- | --- | 1.36 (0.92-2.02) | 0.12 |
| rs3740938 | Codominant | G/G | 190 (61.9%) | 178 (59.3%) | 1 | 0.81 |
|  |  | G/A | 103 (33.5%) | 108 (36%) | 1.12 (0.80-1.57) |  |
|  |  | A/A | 14 (4.6%) | 14 (4.7%) | 1.07 (0.49-2.30) |  |
|  | Dominant | G/G | 190 (61.9%) | 178 (59.3%) | 1 | 0.52 |
|  |  | G/A-A/A | 117 (38.1%) | 122 (40.7%) | 1.11 (0.80-1.54) |  |
|  | Recessive | G/G-G/A | 293 (95.4%) | 286 (95.3%) | 1 | 0.95 |
|  |  | A/A | 14 (4.6%) | 14 (4.7%) | 1.02 (0.48-2.19) |  |
|  | Overdominant | G/G-A/A | 204 (66.5%) | 192 (64%) | 1 | 0.53 |
|  |  | G/A | 103 (33.5%) | 108 (36%) | 1.11 (0.80-1.56) |  |
|  | Log-additive | --- | --- | --- | 1.08 (0.82-1.42) | 0.57 |
| rs2012390 | Codominant | A/A | 176 (57.1%) | 165 (55.2%) | 1 | 0.88 |
|  |  | A/G | 113 (36.7%) | 114 (38.1%) | 1.08 (0.77-1.51) |  |
|  |  | G/G | 19 (6.2%) | 20 (6.7%) | 1.12 (0.58-2.18) |  |
|  | Dominant | A/A | 176 (57.1%) | 165 (55.2%) | 1 | 0.63 |
|  |  | A/G-G/G | 132 (42.9%) | 134 (44.8%) | 1.08 (0.79-1.49) |  |
|  | Recessive | A/A-A/G | 289 (93.8%) | 279 (93.3%) | 1 | 0.79 |
|  |  | G/G | 19 (6.2%) | 20 (6.7%) | 1.09 (0.57-2.09) |  |
|  | Overdominant | A/A-G/G | 195 (63.3%) | 185 (61.9%) | 1 | 0.71 |
|  |  | A/G | 113 (36.7%) | 114 (38.1%) | 1.06 (0.77-1.48) |  |
|  | Log-additive | --- | --- | --- | 1.07 (0.82-1.38) | 0.62 |
| rs1940475 | Codominant | C/C | 129 (41.9%) | 125 (41.7%) | 1 | 0.52 |
|  |  | C/T | 144 (46.8%) | 132 (44%) | 0.95 (0.67-1.33) |  |
|  |  | T/T | 35 (11.4%) | 43 (14.3%) | 1.27 (0.76-2.11) |  |
|  | Dominant | C/C | 129 (41.9%) | 125 (41.7%) | 1 | 0.96 |
|  |  | C/T-T/T | 179 (58.1%) | 175 (58.3%) | 1.01 (0.73-1.39) |  |
|  | Recessive | C/C-C/T | 273 (88.6%) | 257 (85.7%) | 1 | 0.27 |
|  |  | T/T | 35 (11.4%) | 43 (14.3%) | 1.31 (0.81-2.10) |  |
|  | Overdominant | C/C-T/T | 164 (53.2%) | 168 (56%) | 1 | 0.5 |
|  |  | C/T | 144 (46.8%) | 132 (44%) | 0.89 (0.65-1.23) |  |
|  | Log-additive | --- | --- | --- | 1.07 (0.85-1.35) | 0.56 |
| rs11225394 | --- | C/C | 238 (81.8%) | 235 (78.3%) | 1 | 0.29 |
|  |  | T/C | 53 (18.2%) | 65 (21.7%) | 1.24 (0.83-1.86) |  |
| rs11225395 | Codominant | G/G | 131 (42.5%) | 129 (43%) | 1 | 0.63 |
|  |  | A/G | 143 (46.4%) | 131 (43.7%) | 0.93 (0.66-1.31) |  |
|  |  | A/A | 34 (11%) | 40 (13.3%) | 1.19 (0.71-2.01) |  |
|  | Dominant | G/G | 131 (42.5%) | 129 (43%) | 1 | 0.91 |
|  |  | A/G-A/A | 177 (57.5%) | 171 (57%) | 0.98 (0.71-1.35) |  |
|  | Recessive | G/G-A/G | 274 (89%) | 260 (86.7%) | 1 | 0.39 |
|  |  | A/A | 34 (11%) | 40 (13.3%) | 1.24 (0.76-2.02) |  |
|  | Overdominant | G/G-A/A | 165 (53.6%) | 169 (56.3%) | 1 | 0.49 |
|  |  | A/G | 143 (46.4%) | 131 (43.7%) | 0.89 (0.65-1.23) |  |
|  | Log-additive | --- | --- | --- | 1.04 (0.82-1.32) | 0.74 |
| rs639752 | Codominant | A/A | 130 (42.2%) | 139 (46.3%) | 1 | 0.54 |
|  |  | C/A | 146 (47.4%) | 135 (45%) | 0.86 (0.62-1.21) |  |
|  |  | C/C | 32 (10.4%) | 26 (8.7%) | 0.76 (0.43-1.34) |  |
|  | Dominant | A/A | 130 (42.2%) | 139 (46.3%) | 1 | 0.31 |
|  |  | C/A-C/C | 178 (57.8%) | 161 (53.7%) | 0.85 (0.61-1.17) |  |
|  | Recessive | A/A-C/A | 276 (89.6%) | 274 (91.3%) | 1 | 0.47 |
|  |  | C/C | 32 (10.4%) | 26 (8.7%) | 0.82 (0.48-1.41) |  |
|  | Overdominant | A/A-C/C | 162 (52.6%) | 165 (55%) | 1 | 0.55 |
|  |  | C/A | 146 (47.4%) | 135 (45%) | 0.91 (0.66-1.25) |  |
|  | Log-additive | --- | --- | --- | 0.87 (0.68-1.11) | 0.26 |
| rs650108 | Codominant | A/A | 94 (30.6%) | 112 (37.5%) | 1 | 0.16 |
|  |  | A/G | 166 (54.1%) | 140 (46.8%) | 0.71 (0.50-1.01) |  |
|  |  | G/G | 47 (15.3%) | 47 (15.7%) | 0.84 (0.51-1.37) |  |
|  | Dominant | A/A | 94 (30.6%) | 112 (37.5%) | 1 | 0.075 |
|  |  | A/G-G/G | 213 (69.4%) | 187 (62.5%) | 0.74 (0.53-1.03) |  |
|  | Recessive | A/A-A/G | 260 (84.7%) | 252 (84.3%) | 1 | 0.89 |
|  |  | G/G | 47 (15.3%) | 47 (15.7%) | 1.03 (0.66-1.60) |  |
|  | Overdominant | A/A-G/G | 141 (45.9%) | 159 (53.2%) | 1 | 0.074 |
|  |  | A/G | 166 (54.1%) | 140 (46.8%) | 0.75 (0.54-1.03) |  |
|  | Log-additive | --- | --- | --- | 0.87 (0.69-1.10) | 0.24 |
| rs520540 | Codominant | G/G | 130 (42.2%) | 139 (46.3%) | 1 | 0.54 |
|  |  | A/G | 146 (47.4%) | 135 (45%) | 0.86 (0.62-1.21) |  |
|  |  | A/A | 32 (10.4%) | 26 (8.7%) | 0.76 (0.43-1.34) |  |
|  | Dominant | G/G | 130 (42.2%) | 139 (46.3%) | 1 | 0.31 |
|  |  | A/G-A/A | 178 (57.8%) | 161 (53.7%) | 0.85 (0.61-1.17) |  |
|  | Recessive | G/G-A/G | 276 (89.6%) | 274 (91.3%) | 1 | 0.47 |
|  |  | A/A | 32 (10.4%) | 26 (8.7%) | 0.82 (0.48-1.41) |  |
|  | Overdominant | G/G-A/A | 162 (52.6%) | 165 (55%) | 1 | 0.55 |
|  |  | A/G | 146 (47.4%) | 135 (45%) | 0.91 (0.66-1.25) |  |
|  | Log-additive | --- | --- | --- | 0.87 (0.68-1.11) | 0.26 |
| rs646910 | Codominant | T/T | 260 (84.4%) | 256 (85.3%) | 1 | 0.95 |
|  |  | A/T | 46 (14.9%) | 42 (14%) | 0.93 (0.59-1.46) |  |
|  |  | A/A | 2 (0.6%) | 2 (0.7%) | 1.02 (0.14-7.27) |  |
|  | Dominant | T/T | 260 (84.4%) | 256 (85.3%) | 1 | 0.75 |
|  |  | A/T-A/A | 48 (15.6%) | 44 (14.7%) | 0.93 (0.60-1.45) |  |
|  | Recessive | T/T-A/T | 306 (99.3%) | 298 (99.3%) | 1 | 0.98 |
|  |  | A/A | 2 (0.6%) | 2 (0.7%) | 1.03 (0.14-7.34) |  |
|  | Overdominant | T/T-A/A | 262 (85.1%) | 258 (86%) | 1 | 0.74 |
|  |  | A/T | 46 (14.9%) | 42 (14%) | 0.93 (0.59-1.46) |  |
|  | Log-additive | --- | --- | --- | 0.94 (0.62-1.43) | 0.77 |
| rs602128 | Codominant | G/G | 130 (42.6%) | 137 (45.8%) | 1 | 0.63 |
|  |  | G/A | 143 (46.9%) | 136 (45.5%) | 0.90 (0.65-1.26) |  |
|  |  | A/A | 32 (10.5%) | 26 (8.7%) | 0.77 (0.44-1.36) |  |
|  | Dominant | G/G | 130 (42.6%) | 137 (45.8%) | 1 | 0.43 |
|  |  | G/A-A/A | 175 (57.4%) | 162 (54.2%) | 0.88 (0.64-1.21) |  |
|  | Recessive | G/G-G/A | 273 (89.5%) | 273 (91.3%) | 1 | 0.45 |
|  |  | A/A | 32 (10.5%) | 26 (8.7%) | 0.81 (0.47-1.40) |  |
|  | Overdominant | G/G-A/A | 162 (53.1%) | 163 (54.5%) | 1 | 0.73 |
|  |  | G/A | 143 (46.9%) | 136 (45.5%) | 0.95 (0.69-1.30) |  |
|  | Log-additive | --- | --- | --- | 0.89 (0.69-1.14) | 0.34 |
| rs679620 | Codominant | C/C | 130 (42.2%) | 138 (46%) | 1 | 0.53 |
|  |  | T/C | 145 (47.1%) | 136 (45.3%) | 0.88 (0.63-1.23) |  |
|  |  | T/T | 33 (10.7%) | 26 (8.7%) | 0.74 (0.42-1.31) |  |
|  | Dominant | C/C | 130 (42.2%) | 138 (46%) | 1 | 0.35 |
|  |  | T/C-T/T | 178 (57.8%) | 162 (54%) | 0.86 (0.62-1.18) |  |
|  | Recessive | C/C-T/C | 275 (89.3%) | 274 (91.3%) | 1 | 0.39 |
|  |  | T/T | 33 (10.7%) | 26 (8.7%) | 0.79 (0.46-1.36) |  |
|  | Overdominant | C/C-T/T | 163 (52.9%) | 164 (54.7%) | 1 | 0.67 |
|  |  | T/C | 145 (47.1%) | 136 (45.3%) | 0.93 (0.68-1.28) |  |
|  | Log-additive | --- | --- | --- | 0.87 (0.68-1.11) | 0.27 |
| rs678815 | Codominant | C/C | 130 (42.2%) | 137 (46.1%) | 1 | 0.57 |
|  |  | C/G | 146 (47.4%) | 134 (45.1%) | 0.87 (0.62-1.22) |  |
|  |  | G/G | 32 (10.4%) | 26 (8.8%) | 0.77 (0.44-1.36) |  |
|  | Dominant | C/C | 130 (42.2%) | 137 (46.1%) | 1 | 0.33 |
|  |  | C/G-G/G | 178 (57.8%) | 160 (53.9%) | 0.85 (0.62-1.18) |  |
|  | Recessive | C/C-C/G | 276 (89.6%) | 271 (91.2%) | 1 | 0.49 |
|  |  | G/G | 32 (10.4%) | 26 (8.8%) | 0.83 (0.48-1.43) |  |
|  | Overdominant | C/C-G/G | 162 (52.6%) | 163 (54.9%) | 1 | 0.57 |
|  |  | C/G | 146 (47.4%) | 134 (45.1%) | 0.91 (0.66-1.26) |  |
|  | Log-additive | --- | --- | --- | 0.88 (0.68-1.12) | 0.29 |
| rs522616 | Codominant | T/T | 123 (40.1%) | 113 (37.7%) | 1 | 0.5 |
|  |  | T/C | 144 (46.9%) | 138 (46%) | 1.04 (0.74-1.47) |  |
|  |  | C/C | 40 (13%) | 49 (16.3%) | 1.33 (0.82-2.18) |  |
|  | Dominant | T/T | 123 (40.1%) | 113 (37.7%) | 1 | 0.54 |
|  |  | T/C-C/C | 184 (59.9%) | 187 (62.3%) | 1.11 (0.80-1.53) |  |
|  | Recessive | T/T-T/C | 267 (87%) | 251 (83.7%) | 1 | 0.25 |
|  |  | C/C | 40 (13%) | 49 (16.3%) | 1.30 (0.83-2.05) |  |
|  | Overdominant | T/T-C/C | 163 (53.1%) | 162 (54%) | 1 | 0.82 |
|  |  | T/C | 144 (46.9%) | 138 (46%) | 0.96 (0.70-1.33) |  |
|  | Log-additive | --- | --- | --- | 1.13 (0.89-1.42) | 0.31 |
