# Supplementary material for: Association between alcohol-induced osteonecrosis of femoral head and risk variants of MMPS in Han population based on a case-control study
Source: Oncotarget. 2017 Mar 18;8(38):64490–8. doi: 10.18632/oncotarget.16380 (PMC5610020; doi:10.18632/oncotarget.16380)
Supplement: Supplementary file 3 [file oncotarget-08-64490-s003.docx]

Association between alcohol-induced osteonecrosis of femoral head and risk variants of *MMPS* in Han population based on a case-control study

**Supplementary Material**

Supplementary Table 3. Logistic analyses of SNPs in all research gene with adjustment Gender and Age.

| SNP | Model | Genotype | Group=control | Group=Alcohol | OR (95% CI) | P-value |
| --- | --- | --- | --- | --- | --- | --- |
| rs14983 | Codominant | G/G | 167 (54.2%) | 158 (52.7%) | 1 | 0.8 |
|  |  | G/A | 121 (39.3%) | 122 (40.7%) | 1.10 (0.74-1.63) |  |
|  |  | A/A | 20 (6.5%) | 20 (6.7%) | 1.25 (0.59-2.65) |  |
|  | Dominant | G/G | 167 (54.2%) | 158 (52.7%) | 1 | 0.56 |
|  |  | G/A-A/A | 141 (45.8%) | 142 (47.3%) | 1.12 (0.77-1.64) |  |
|  | Recessive | G/G-G/A | 288 (93.5%) | 280 (93.3%) | 1 | 0.63 |
|  |  | A/A | 20 (6.5%) | 20 (6.7%) | 1.20 (0.57-2.50) |  |
|  | Overdominant | G/G-A/A | 187 (60.7%) | 178 (59.3%) | 1 | 0.73 |
|  |  | G/A | 121 (39.3%) | 122 (40.7%) | 1.07 (0.73-1.57) |  |
|  | Log-additive | --- | --- | --- | 1.11 (0.82-1.50) | 0.51 |
| rs17352054 | Codominant | A/A | 235 (76.3%) | 227 (75.7%) | 1 | 0.74 |
|  |  | C/A | 68 (22.1%) | 68 (22.7%) | 1.09 (0.69-1.71) |  |
|  |  | C/C | 5 (1.6%) | 5 (1.7%) | 1.85 (0.33-10.52) |  |
|  | Dominant | A/A | 235 (76.3%) | 227 (75.7%) | 1 | 0.62 |
|  |  | C/A-C/C | 73 (23.7%) | 73 (24.3%) | 1.12 (0.72-1.75) |  |
|  | Recessive | A/A-C/A | 303 (98.4%) | 295 (98.3%) | 1 | 0.49 |
|  |  | C/C | 5 (1.6%) | 5 (1.7%) | 1.82 (0.32-10.29) |  |
|  | Overdominant | A/A-C/C | 240 (77.9%) | 232 (77.3%) | 1 | 0.76 |
|  |  | C/A | 68 (22.1%) | 68 (22.7%) | 1.07 (0.68-1.69) |  |
|  | Log-additive | --- | --- | --- | 1.14 (0.76-1.72) | 0.54 |
| rs10502001 | Codominant | C/C | 166 (54.1%) | 158 (52.7%) | 1 | 0.82 |
|  |  | T/C | 121 (39.4%) | 122 (40.7%) | 1.09 (0.73-1.62) |  |
|  |  | T/T | 20 (6.5%) | 20 (6.7%) | 1.23 (0.58-2.63) |  |
|  | Dominant | C/C | 166 (54.1%) | 158 (52.7%) | 1 | 0.59 |
|  |  | T/C-T/T | 141 (45.9%) | 142 (47.3%) | 1.11 (0.76-1.62) |  |
|  | Recessive | C/C-T/C | 287 (93.5%) | 280 (93.3%) | 1 | 0.64 |
|  |  | T/T | 20 (6.5%) | 20 (6.7%) | 1.19 (0.57-2.48) |  |
|  | Overdominant | C/C-T/T | 186 (60.6%) | 178 (59.3%) | 1 | 0.76 |
|  |  | T/C | 121 (39.4%) | 122 (40.7%) | 1.06 (0.72-1.56) |  |
|  | Log-additive | --- | --- | --- | 1.10 (0.81-1.49) | 0.53 |
| rs11568818 | Codominant | T/T | 262 (85.1%) | 242 (80.7%) | 1 | 0.85 |
|  |  | C/T | 44 (14.3%) | 54 (18%) | 1.04 (0.63-1.71) |  |
|  |  | C/C | 2 (0.6%) | 4 (1.3%) | 1.82 (0.21-16.13) |  |
|  | Dominant | T/T | 262 (85.1%) | 242 (80.7%) | 1 | 0.81 |
|  |  | C/T-C/C | 46 (14.9%) | 58 (19.3%) | 1.06 (0.65-1.74) |  |
|  | Recessive | T/T-C/T | 306 (99.3%) | 296 (98.7%) | 1 | 0.57 |
|  |  | C/C | 2 (0.6%) | 4 (1.3%) | 1.81 (0.20-16.00) |  |
|  | Overdominant | T/T-C/C | 264 (85.7%) | 246 (82%) | 1 | 0.91 |
|  |  | C/T | 44 (14.3%) | 54 (18%) | 1.03 (0.62-1.70) |  |
|  | Log-additive | --- | --- | --- | 1.09 (0.69-1.72) | 0.72 |
| rs17098318 | Codominant | G/G | 263 (85.4%) | 243 (81%) | 1 | 0.85 |
|  |  | A/G | 43 (14%) | 53 (17.7%) | 1.03 (0.62-1.70) |  |
|  |  | A/A | 2 (0.6%) | 4 (1.3%) | 1.82 (0.21-16.10) |  |
|  | Dominant | G/G | 263 (85.4%) | 243 (81%) | 1 | 0.83 |
|  |  | A/G-A/A | 45 (14.6%) | 57 (19%) | 1.05 (0.64-1.73) |  |
|  | Recessive | G/G-A/G | 306 (99.3%) | 296 (98.7%) | 1 | 0.57 |
|  |  | A/A | 2 (0.6%) | 4 (1.3%) | 1.81 (0.20-16.00) |  |
|  | Overdominant | G/G-A/A | 265 (86%) | 247 (82.3%) | 1 | 0.94 |
|  |  | A/G | 43 (14%) | 53 (17.7%) | 1.02 (0.62-1.69) |  |
|  | Log-additive | --- | --- | --- | 1.08 (0.68-1.70) | 0.75 |
| rs3740938 | Codominant | G/G | 190 (61.9%) | 178 (59.3%) | 1 | 0.59 |
|  |  | G/A | 103 (33.5%) | 108 (36%) | 0.82 (0.55-1.22) |  |
|  |  | A/A | 14 (4.6%) | 14 (4.7%) | 0.81 (0.32-2.00) |  |
|  | Dominant | G/G | 190 (61.9%) | 178 (59.3%) | 1 | 0.31 |
|  |  | G/A-A/A | 117 (38.1%) | 122 (40.7%) | 0.82 (0.56-1.20) |  |
|  | Recessive | G/G-G/A | 293 (95.4%) | 286 (95.3%) | 1 | 0.77 |
|  |  | A/A | 14 (4.6%) | 14 (4.7%) | 0.87 (0.36-2.14) |  |
|  | Overdominant | G/G-A/A | 204 (66.5%) | 192 (64%) | 1 | 0.36 |
|  |  | G/A | 103 (33.5%) | 108 (36%) | 0.84 (0.57-1.23) |  |
|  | Log-additive | --- | --- | --- | 0.85 (0.62-1.18) | 0.33 |
| rs2012390 | Codominant | A/A | 176 (57.1%) | 165 (55.2%) | 1 | 0.52 |
|  |  | A/G | 113 (36.7%) | 114 (38.1%) | 0.80 (0.54-1.18) |  |
|  |  | G/G | 19 (6.2%) | 20 (6.7%) | 0.89 (0.41-1.93) |  |
|  | Dominant | A/A | 176 (57.1%) | 165 (55.2%) | 1 | 0.27 |
|  |  | A/G-G/G | 132 (42.9%) | 134 (44.8%) | 0.81 (0.55-1.18) |  |
|  | Recessive | A/A-A/G | 289 (93.8%) | 279 (93.3%) | 1 | 0.97 |
|  |  | G/G | 19 (6.2%) | 20 (6.7%) | 0.98 (0.46-2.09) |  |
|  | Overdominant | A/A-G/G | 195 (63.3%) | 185 (61.9%) | 1 | 0.27 |
|  |  | A/G | 113 (36.7%) | 114 (38.1%) | 0.81 (0.55-1.18) |  |
|  | Log-additive | --- | --- | --- | 0.87 (0.64-1.18) | 0.37 |
| rs1940475 | Codominant | C/C | 129 (41.9%) | 125 (41.7%) | 1 | 0.66 |
|  |  | C/T | 144 (46.8%) | 132 (44%) | 0.86 (0.57-1.30) |  |
|  |  | T/T | 35 (11.4%) | 43 (14.3%) | 1.08 (0.60-1.95) |  |
|  | Dominant | C/C | 129 (41.9%) | 125 (41.7%) | 1 | 0.63 |
|  |  | C/T-T/T | 179 (58.1%) | 175 (58.3%) | 0.91 (0.62-1.34) |  |
|  | Recessive | C/C-C/T | 273 (88.6%) | 257 (85.7%) | 1 | 0.57 |
|  |  | T/T | 35 (11.4%) | 43 (14.3%) | 1.17 (0.68-2.02) |  |
|  | Overdominant | C/C-T/T | 164 (53.2%) | 168 (56%) | 1 | 0.38 |
|  |  | C/T | 144 (46.8%) | 132 (44%) | 0.84 (0.58-1.24) |  |
|  | Log-additive | --- | --- | --- | 0.99 (0.76-1.30) | 0.96 |
| rs11225394 | --- | C/C | 238 (81.8%) | 235 (78.3%) | 1 | 0.047 |
|  |  | T/C | 53 (18.2%) | 65 (21.7%) | 1.64 (1.00-2.70) |  |
| rs11225395 | Codominant | G/G | 131 (42.5%) | 129 (43%) | 1 | 0.74 |
|  |  | A/G | 143 (46.4%) | 131 (43.7%) | 0.87 (0.58-1.30) |  |
|  |  | A/A | 34 (11%) | 40 (13.3%) | 1.03 (0.57-1.87) |  |
|  | Dominant | G/G | 131 (42.5%) | 129 (43%) | 1 | 0.59 |
|  |  | A/G-A/A | 177 (57.5%) | 171 (57%) | 0.90 (0.61-1.32) |  |
|  | Recessive | G/G-A/G | 274 (89%) | 260 (86.7%) | 1 | 0.72 |
|  |  | A/A | 34 (11%) | 40 (13.3%) | 1.11 (0.63-1.94) |  |
|  | Overdominant | G/G-A/A | 165 (53.6%) | 169 (56.3%) | 1 | 0.44 |
|  |  | A/G | 143 (46.4%) | 131 (43.7%) | 0.86 (0.59-1.26) |  |
|  | Log-additive | --- | --- | --- | 0.97 (0.74-1.28) | 0.84 |
| rs639752 | Codominant | A/A | 130 (42.2%) | 139 (46.3%) | 1 | 0.37 |
|  |  | C/A | 146 (47.4%) | 135 (45%) | 0.79 (0.53-1.18) |  |
|  |  | C/C | 32 (10.4%) | 26 (8.7%) | 0.69 (0.36-1.30) |  |
|  | Dominant | A/A | 130 (42.2%) | 139 (46.3%) | 1 | 0.18 |
|  |  | C/A-C/C | 178 (57.8%) | 161 (53.7%) | 0.77 (0.52-1.13) |  |
|  | Recessive | A/A-C/A | 276 (89.6%) | 274 (91.3%) | 1 | 0.41 |
|  |  | C/C | 32 (10.4%) | 26 (8.7%) | 0.77 (0.42-1.42) |  |
|  | Overdominant | A/A-C/C | 162 (52.6%) | 165 (55%) | 1 | 0.41 |
|  |  | C/A | 146 (47.4%) | 135 (45%) | 0.85 (0.58-1.24) |  |
|  | Log-additive | --- | --- | --- | 0.82 (0.61-1.09) | 0.16 |
| rs650108 | Codominant | A/A | 94 (30.6%) | 112 (37.5%) | 1 | 0.25 |
|  |  | A/G | 166 (54.1%) | 140 (46.8%) | 0.71 (0.46-1.09) |  |
|  |  | G/G | 47 (15.3%) | 47 (15.7%) | 0.71 (0.41-1.25) |  |
|  | Dominant | A/A | 94 (30.6%) | 112 (37.5%) | 1 | 0.094 |
|  |  | A/G-G/G | 213 (69.4%) | 187 (62.5%) | 0.71 (0.47-1.06) |  |
|  | Recessive | A/A-A/G | 260 (84.7%) | 252 (84.3%) | 1 | 0.61 |
|  |  | G/G | 47 (15.3%) | 47 (15.7%) | 0.88 (0.53-1.44) |  |
|  | Overdominant | A/A-G/G | 141 (45.9%) | 159 (53.2%) | 1 | 0.24 |
|  |  | A/G | 166 (54.1%) | 140 (46.8%) | 0.80 (0.55-1.16) |  |
|  | Log-additive | --- | --- | --- | 0.82 (0.63-1.08) | 0.16 |
| rs520540 | Codominant | G/G | 130 (42.2%) | 139 (46.3%) | 1 | 0.37 |
|  |  | A/G | 146 (47.4%) | 135 (45%) | 0.79 (0.53-1.18) |  |
|  |  | A/A | 32 (10.4%) | 26 (8.7%) | 0.69 (0.36-1.30) |  |
|  | Dominant | G/G | 130 (42.2%) | 139 (46.3%) | 1 | 0.18 |
|  |  | A/G-A/A | 178 (57.8%) | 161 (53.7%) | 0.77 (0.52-1.13) |  |
|  | Recessive | G/G-A/G | 276 (89.6%) | 274 (91.3%) | 1 | 0.41 |
|  |  | A/A | 32 (10.4%) | 26 (8.7%) | 0.77 (0.42-1.42) |  |
|  | Overdominant | G/G-A/A | 162 (52.6%) | 165 (55%) | 1 | 0.41 |
|  |  | A/G | 146 (47.4%) | 135 (45%) | 0.85 (0.58-1.24) |  |
|  | Log-additive | --- | --- | --- | 0.82 (0.61-1.09) | 0.16 |
| rs646910 | Codominant | T/T | 260 (84.4%) | 256 (85.3%) | 1 | 0.88 |
|  |  | A/T | 46 (14.9%) | 42 (14%) | 0.89 (0.52-1.50) |  |
|  |  | A/A | 2 (0.6%) | 2 (0.7%) | 1.31 (0.12-14.65) |  |
|  | Dominant | T/T | 260 (84.4%) | 256 (85.3%) | 1 | 0.69 |
|  |  | A/T-A/A | 48 (15.6%) | 44 (14.7%) | 0.90 (0.54-1.51) |  |
|  | Recessive | T/T-A/T | 306 (99.3%) | 298 (99.3%) | 1 | 0.81 |
|  |  | A/A | 2 (0.6%) | 2 (0.7%) | 1.34 (0.12-14.90) |  |
|  | Overdominant | T/T-A/A | 262 (85.1%) | 258 (86%) | 1 | 0.64 |
|  |  | A/T | 46 (14.9%) | 42 (14%) | 0.88 (0.52-1.49) |  |
|  | Log-additive | --- | --- | --- | 0.92 (0.57-1.50) | 0.74 |
| rs602128 | Codominant | G/G | 130 (42.6%) | 137 (45.8%) | 1 | 0.45 |
|  |  | G/A | 143 (46.9%) | 136 (45.5%) | 0.83 (0.55-1.24) |  |
|  |  | A/A | 32 (10.5%) | 26 (8.7%) | 0.70 (0.37-1.32) |  |
|  | Dominant | G/G | 130 (42.6%) | 137 (45.8%) | 1 | 0.26 |
|  |  | G/A-A/A | 175 (57.4%) | 162 (54.2%) | 0.80 (0.55-1.18) |  |
|  | Recessive | G/G-G/A | 273 (89.5%) | 273 (91.3%) | 1 | 0.39 |
|  |  | A/A | 32 (10.5%) | 26 (8.7%) | 0.77 (0.42-1.41) |  |
|  | Overdominant | G/G-A/A | 162 (53.1%) | 163 (54.5%) | 1 | 0.55 |
|  |  | G/A | 143 (46.9%) | 136 (45.5%) | 0.89 (0.61-1.30) |  |
|  | Log-additive | --- | --- | --- | 0.83 (0.62-1.11) | 0.21 |
| rs679620 | Codominant | C/C | 130 (42.2%) | 138 (46%) | 1 | 0.37 |
|  |  | T/C | 145 (47.1%) | 136 (45.3%) | 0.81 (0.54-1.21) |  |
|  |  | T/T | 33 (10.7%) | 26 (8.7%) | 0.67 (0.35-1.26) |  |
|  | Dominant | C/C | 130 (42.2%) | 138 (46%) | 1 | 0.2 |
|  |  | T/C-T/T | 178 (57.8%) | 162 (54%) | 0.78 (0.53-1.14) |  |
|  | Recessive | C/C-T/C | 275 (89.3%) | 274 (91.3%) | 1 | 0.33 |
|  |  | T/T | 33 (10.7%) | 26 (8.7%) | 0.74 (0.41-1.35) |  |
|  | Overdominant | C/C-T/T | 163 (52.9%) | 164 (54.7%) | 1 | 0.51 |
|  |  | T/C | 145 (47.1%) | 136 (45.3%) | 0.88 (0.60-1.29) |  |
|  | Log-additive | --- | --- | --- | 0.81 (0.61-1.08) | 0.16 |
| rs678815 | Codominant | C/C | 130 (42.2%) | 137 (46.1%) | 1 | 0.39 |
|  |  | C/G | 146 (47.4%) | 134 (45.1%) | 0.80 (0.53-1.19) |  |
|  |  | G/G | 32 (10.4%) | 26 (8.8%) | 0.69 (0.36-1.32) |  |
|  | Dominant | C/C | 130 (42.2%) | 137 (46.1%) | 1 | 0.19 |
|  |  | C/G-G/G | 178 (57.8%) | 160 (53.9%) | 0.78 (0.53-1.14) |  |
|  | Recessive | C/C-C/G | 276 (89.6%) | 271 (91.2%) | 1 | 0.42 |
|  |  | G/G | 32 (10.4%) | 26 (8.8%) | 0.78 (0.42-1.43) |  |
|  | Overdominant | C/C-G/G | 162 (52.6%) | 163 (54.9%) | 1 | 0.43 |
|  |  | C/G | 146 (47.4%) | 134 (45.1%) | 0.86 (0.59-1.26) |  |
|  | Log-additive | --- | --- | --- | 0.82 (0.62-1.09) | 0.18 |
| rs522616 | Codominant | T/T | 123 (40.1%) | 113 (37.7%) | 1 | 0.22 |
|  |  | T/C | 144 (46.9%) | 138 (46%) | 1.05 (0.70-1.58) |  |
|  |  | C/C | 40 (13%) | 49 (16.3%) | 1.66 (0.91-3.03) |  |
|  | Dominant | T/T | 123 (40.1%) | 113 (37.7%) | 1 | 0.43 |
|  |  | T/C-C/C | 184 (59.9%) | 187 (62.3%) | 1.17 (0.79-1.72) |  |
|  | Recessive | T/T-T/C | 267 (87%) | 251 (83.7%) | 1 | 0.085 |
|  |  | C/C | 40 (13%) | 49 (16.3%) | 1.62 (0.93-2.82) |  |
|  | Overdominant | T/T-C/C | 163 (53.1%) | 162 (54%) | 1 | 0.66 |
|  |  | T/C | 144 (46.9%) | 138 (46%) | 0.92 (0.63-1.34) |  |
|  | Log-additive | --- | --- | --- | 1.22 (0.93-1.61) | 0.15 |
| rs1053605 | Codominant | C/C | 242 (78.6%) | 232 (77.3%) | 1 | 0.44 |
|  |  | C/T | 63 (20.4%) | 62 (20.7%) | 0.82 (0.52-1.30) |  |
|  |  | T/T | 3 (1%) | 6 (2%) | 2.14 (0.39-11.73) |  |
|  | Dominant | C/C | 242 (78.6%) | 232 (77.3%) | 1 | 0.54 |
|  |  | C/T-T/T | 66 (21.4%) | 68 (22.7%) | 0.87 (0.56-1.36) |  |
|  | Recessive | C/C-C/T | 305 (99%) | 294 (98%) | 1 | 0.33 |
|  |  | T/T | 3 (1%) | 6 (2%) | 2.23 (0.41-12.18) |  |
|  | Overdominant | C/C-T/T | 245 (79.5%) | 238 (79.3%) | 1 | 0.37 |
|  |  | C/T | 63 (20.4%) | 62 (20.7%) | 0.81 (0.51-1.28) |  |
|  | Log-additive | --- | --- | --- | 0.94 (0.63-1.41) | 0.76 |
| rs243849 | Codominant | C/C | 216 (70.1%) | 191 (63.7%) | 1 | 0.15 |
|  |  | C/T | 83 (26.9%) | 92 (30.7%) | 1.43 (0.93-2.18) |  |
|  |  | T/T | 9 (2.9%) | 17 (5.7%) | 1.87 (0.69-5.09) |  |
|  | Dominant | C/C | 216 (70.1%) | 191 (63.7%) | 1 | 0.06 |
|  |  | C/T-T/T | 92 (29.9%) | 109 (36.3%) | 1.47 (0.98-2.22) |  |
|  | Recessive | C/C-C/T | 299 (97.1%) | 283 (94.3%) | 1 | 0.29 |
|  |  | T/T | 9 (2.9%) | 17 (5.7%) | 1.68 (0.63-4.54) |  |
|  | Overdominant | C/C-T/T | 225 (73%) | 208 (69.3%) | 1 | 0.14 |
|  |  | C/T | 83 (26.9%) | 92 (30.7%) | 1.38 (0.90-2.10) |  |
|  | Log-additive | --- | --- | --- | 1.40 (0.99-1.98) | 0.051 |
| rs243847 | Codominant | T/T | 109 (35.4%) | 118 (39.3%) | 1 | 0.67 |
|  |  | C/T | 140 (45.5%) | 134 (44.7%) | 1.03 (0.68-1.56) |  |
|  |  | C/C | 59 (19.2%) | 48 (16%) | 0.81 (0.46-1.40) |  |
|  | Dominant | T/T | 109 (35.4%) | 118 (39.3%) | 1 | 0.85 |
|  |  | C/T-C/C | 199 (64.6%) | 182 (60.7%) | 0.96 (0.65-1.42) |  |
|  | Recessive | T/T-C/T | 249 (80.8%) | 252 (84%) | 1 | 0.38 |
|  |  | C/C | 59 (19.2%) | 48 (16%) | 0.80 (0.48-1.32) |  |
|  | Overdominant | T/T-C/C | 168 (54.5%) | 166 (55.3%) | 1 | 0.63 |
|  |  | C/T | 140 (45.5%) | 134 (44.7%) | 1.10 (0.75-1.61) |  |
|  | Log-additive | --- | --- | --- | 0.92 (0.71-1.20) | 0.56 |
| rs243832 | Codominant | G/G | 122 (39.6%) | 116 (38.7%) | 1 | 0.57 |
|  |  | G/C | 145 (47.1%) | 150 (50%) | 1.15 (0.76-1.72) |  |
|  |  | C/C | 41 (13.3%) | 34 (11.3%) | 0.84 (0.46-1.55) |  |
|  | Dominant | G/G | 122 (39.6%) | 116 (38.7%) | 1 | 0.71 |
|  |  | G/C-C/C | 186 (60.4%) | 184 (61.3%) | 1.08 (0.73-1.58) |  |
|  | Recessive | G/G-G/C | 267 (86.7%) | 266 (88.7%) | 1 | 0.41 |
|  |  | C/C | 41 (13.3%) | 34 (11.3%) | 0.79 (0.44-1.39) |  |
|  | Overdominant | G/G-C/C | 163 (52.9%) | 150 (50%) | 1 | 0.36 |
|  |  | G/C | 145 (47.1%) | 150 (50%) | 1.19 (0.82-1.74) |  |
|  | Log-additive | --- | --- | --- | 0.98 (0.74-1.30) | 0.89 |
| rs7201 | Codominant | A/A | 173 (56.4%) | 178 (59.3%) | 1 | 0.67 |
|  |  | C/A | 116 (37.8%) | 110 (36.7%) | 0.99 (0.67-1.48) |  |
|  |  | C/C | 18 (5.9%) | 12 (4%) | 0.65 (0.26-1.66) |  |
|  | Dominant | A/A | 173 (56.4%) | 178 (59.3%) | 1 | 0.79 |
|  |  | C/A-C/C | 134 (43.6%) | 122 (40.7%) | 0.95 (0.65-1.39) |  |
|  | Recessive | A/A-C/A | 289 (94.1%) | 288 (96%) | 1 | 0.37 |
|  |  | C/C | 18 (5.9%) | 12 (4%) | 0.66 (0.26-1.64) |  |
|  | Overdominant | A/A-C/C | 191 (62.2%) | 190 (63.3%) | 1 | 0.91 |
|  |  | C/A | 116 (37.8%) | 110 (36.7%) | 1.02 (0.69-1.51) |  |
|  | Log-additive | --- | --- | --- | 0.91 (0.66-1.27) | 0.59 |
